# Effects of acute GLP-1 analogue infusion on the glycemic and neurohormonal responses to meal test in non- hypoglycemic subjects after gastric bypass

**DOI:** 10.1007/s12020-025-04446-x

**Published:** 2025-10-10

**Authors:** Kristina E. Almby, Urban Wiklund, Martin H. Lundqvist, Maria J. Pereira, Niclas Abrahamsson

**Affiliations:** 1 Department of Medical Sciences, Clinical Diabetes and Metabolism, Uppsala University, Uppsala, 751 85 Sweden; 2 Department of Diagnostics and Intervention, Biomedical Engineering, Umeå University, Umeå, Sweden

**Keywords:** Roux-en y gastric bypass, Post-bariatric hypoglycaemia, GLP-1 analogue, Hypoglycaemia counterregulation, Cortisol

## Abstract

**Purpose:**

The aim of this study was to assess the effects of acute Glucagon-like peptide-1 (GLP-1) receptor agonist administration on metabolic and endocrine responses to a standardized meal test in individuals who have undergone Roux-en-Y gastric bypass surgery (RYGB), and thus to uncover mechanisms that might be important in post-bariatric hypoglycemia.

**Methods:**

In a single-center, randomized crossover study, 10 patients without diabetes underwent two standardized high-carbohydrate meal tests with either saline (SAL) or exenatide (EXE) infusion, one year after RYGB. We assessed glucose, insulin, c-peptide, ACTH, cortisol, GH, adrenalin, noradrenalin, dopamine, glucagon, active GLP-1 and GIP. Gastric emptying rate was estimated with repeated paracetamol measurements. Heart rate variability was recorded as a marker of sympathetic nervous system activity.

**Results:**

Postprandial glucose levels dropped below baseline levels in all subjects. Cortisol levels were higher during EXE infusion. As expected, insulin and c-peptide levels were higher with EXE. No other significant differences were observed in the measured hormones. EXE did not affect gastric emptying rate. HRV showed a decreased RR-interval during EXE infusion.

**Conclusion:**

During a meal test, acute exenatide infusion led to elevated cortisol levels but did not protect against post-prandial drops in glucose levels. Taken together with previous research, this implicates that alleviation of post-prandial hypoglycemia after RYGB via GLP-1 receptor agonist treatment likely involves other mechanisms than acute effects on conventional counter-regulatory hormones.

## Introduction

Roux-en-Y gastric bypass (RYGB) surgery is one of the most effective treatments for morbid obesity. Several changes occur after surgery, perhaps most importantly a swift improvement in glycemic control, frequently leading to fast remission of diabetes mellitus type 2 [1, 2]. Post-surgery, the release of intestinal hormone Glucagon-like peptide-1 (GLP-1) is increased approximately 10-fold in response to a meal [3], which is considered responsible for the main part of the increased satiety, weight-loss, and the improved prandial insulin response post-surgery [4].

One potential and severe complication of RYGB is post-bariatric postprandial hypoglycemia (PBH); the tendency to develop pathologically low blood sugar after food intake. Also known as hyperinsulinemic hypoglycaemia [5] or postprandial reactive hypoglycemic syndrome, it is usually differentiated from dumping [6]. While these phenomena partly overlap, dumping symptoms (such as abdominal pain, nausea, bloating and fatigue) typically occur in the first hour after a meal, whereas hypoglycemia usually occurs at a later point, around 1–3 h after food intake.

The mechanism behind PBH is incompletely understood but likely involves a mismatch between rapid glucose uptake and the magnitude and timing of insulin release, as well as improved insulin sensitivity [5, 7, 8]. The surge of post-prandial GLP-1 is thought to contribute [9]. Salehi et al. [8] demonstrated a faster rate of oral glucose appearance in blood and more than double GLP-1 and insulin levels during a mixed meal test (MMT) in subjects with PBH [8].

Competitive GLP-1 antagonist Exendin (9 − 3) has been shown to normalize the exaggerated insulin secretion after MMT and to prevent hypoglycemia during MMT [8] and during oral glucose tolerance test (OGTT) [10]. Our group has previously demonstrated a reduction of hypoglycemic episodes as well as an alleviation of hypoglycemic symptoms during treatment with GLP-1 receptor agonist (GLP-1RA) liraglutide [11], which has been supported by other research [9].

One hypothesized mechanism behind this effect is that GLP-1RA might enhance the counterregulatory response to hypoglycemia. To assess this, our group previously performed stepwise euglycemic-hypoglycemic clamps in patients one year after RYGB and measured the counterregulatory responses to hypoglycemia during infusion of either exenatide (EXE) or saline (SAL) [12]. With this approach, we found no evidence of changes in growth hormone (GH), glucagon, cortisol, adrenocorticotropic hormone (ACTH), adrenalin or noradrenalin levels with EXE infusion. However, changes to heart rate variability (HRV) parameters that could indicate effects of EXE on Autonomic Nervous System (ANS) activity was observed.

In this study, we have performed similar interventions and assessments during a liquid high carbohydrate meal test, which to a greater extent reflects the physiology of real-life postprandial hypoglycemia, in comparison with clamp conditions. The aim was to assess whether GLP-1 receptor activation exerts hypoglycemia-alleviating effects via mechanisms which become apparent in the post-prandial setting. Similar to previous studies, and given the high prevalence of asymptomatic hypoglycemia [13], we included participants without reported problems with PBH, to characterize responses in the typical post-RYGB state. This approach enables an elucidation of the potential role of GLP-1 receptor activation in the PBH-setting as well as with regard to the marked positive effect of RYGB on glucose metabolism.

## Materials and methods

Study participants were consecutively recruited at their 1-year follow-up after RYGB at the Endocrine unit of Uppsala University Hospital, Sweden. Participants were 18–60 years old, without previous or current diabetes and required to have lost 40–80% of their preoperative excess weight. The participants came for two study visits, each involving a liquid high carbohydrate meal test with a concomitant infusion of either SAL or EXE. The order of infusion was randomized and then repeated for the other condition, in a cross-over fashion, at the next visit approximately 1–2 weeks later. During the study visits anthropometric data were obtained and body composition was assessed with TANITA body composition analyzer BC-418 (TANITA Europe B.V., Amsterdam, the Netherlands). Resting blood pressure was measured and resting heart rate variability was recorded for six minutes, the latter with a single channel ECG system, Actiwave Cardio, (CamNtech, Cambridge, UK). Heart Rate Variability (HRV) was continuously recorded.

At the start of the meal test, participants were administered 1.5 g of paracetamol perorally, as a marker for gastric emptying that has been validated with liquid caloric meal [14]. At the same time, they were given a 300-kcal standardized liquid high carbohydrate meal (200 ml Fortimel Jucy 1,5 kcal/ml, Nutricia Nordica, Solna, Sweden) to be consumed within 10 min. Simultaneously, an intravenous infusion of either a 0.9% NaCl-solution at 10 ml/h or a 0.066 pmol/kg/min solution of exenatide (BYETTA, AstraZeneca, Sweden), as previously implemented [12], was commenced. Exenatide was used in the current study to increase the possibility of comparison with these earlier studies, even though it is now more seldomly used in clinical practice than other GLP-1 receptor agonists. Blood samples were drawn at baseline and at minute 15, 30, 45, 60, 75, 90, 105, 120, 150, 180 and 195 for glucose, c-peptide, insulin and paracetamol, and at minute 60, 90, 120, 180, and 195 for GH, ACTH, cortisol, catecholamines, glucagon, Gastric Inhibitory Polypeptide (GIP) and active GLP-1.

Glucose dynamics was measured as Slope up and Slope down, Slope up was defined as change in glucose per minute from baseline glucose to maximum glucose, Slope down was defined as change in glucose per minute from maximum glucose to nadir.

### Biochemical measurements

Baseline venous blood samples were obtained in the morning, after an overnight fast. Basic chemistry and hematological analyses as well as plasma insulin, c-peptide, cortisol, ACTH, GH and paracetamol were performed at the Department of Clinical Chemistry at Uppsala University Hospital. Plasma glucose and paracetamol were quantified with Cobas c 503 while Cobas e 602/801(Roche, Indianapolis, IN, USA) was used for insulin, cortisol and C-peptide. Immulite 2000XPi (Siemens Healthcare Global, Erlangen, Germany) was used to determine GH and ACTH. Adrenaline, noradrenaline and dopamine tests were performed at the Clinical Diabetes and Metabolism Research Laboratory using 3-CAT Research ELISA (#BA E-5600R; LDN, Nordhorn, Germany). Glucagon was determined with ELISA (#10-1271-01; Mercodia, Uppsala, Sweden) while GIP and active GLP-1 were quantified with U-PLEX Metabolic Group 1 (K151ACL-1, Meso Scale Diagnostics, Rockville, MD, USA). Analyses were carried out in line with the manufacturers’ instructions.

### Heart rate variability (HRV)

HRV analysis involves calculations based on the interbeat variations of the RR interval in electrocardiograms (ECG). The duration of this interval varies with heart frequency, but also with respiratory rate, and has been shown to be affected by both sympathetic and parasympathetic nervous system activity. The HRV data extraction method from the ECG recordings has been detailed previously [12]. For this type of analysis, spectral analysis was used, and the mean RR interval was first subtracted before calculation of the total spectral power (PTOT) using Welch’s periodogram method. The power spectrum was divided into different frequency components, where the power of the low frequency (PLF, 0.04–0.15 Hz) and high frequency (PHF, 0.15–0.50 Hz) components are considered as most reflective of ANS activity [15]. All spectral indices were log-transformed before statistical analysis. The HRV analysis was performed using MATLAB (MathWorks, Natick, MA, USA), where the code for the analysis was developed at Umeå University Hospital, Sweden.

### Statistical analysis

Primary outcome of this study was change in counterregulatory hormones between the two tests, EXE and SAL. Area under the curve (AUC) was calculated using the trapezoidal rule. Comparisons between SAL and EXE were made for AUC using Wilcoxon signed rank test for matched pairs. P-values < 0.05 were considered statistically significant. Assuming a 25% difference between EXE and SAL, as previously demonstrated [12], a power calculation arrived at a target sample size of 8 individuals to detect a significant difference between the treatments with 80% power.

HRV indices were first determined in all successive 5-min segments, then the average was calculated over 15 min segments up to 180 min. No detectable differences were noted before 60 min and thus the statistical analysis was only performed for the part between 60 min and 135 min where there was a near linear change during both SAL (increase) and EXE (decline). This segment was analyzed with linear mixed effects model in MATLAB. Subject id was entered as random effect (random intercept model). Fixed effects included condition (SAL/EXE, factor), time and the interaction between condition and time (both continuous covariates). The normality of standardized residuals was verified with the Kolmogorov-Smirnov test.

Microsoft Excel (Microsoft Corporation, Redmond, WA, USA), GraphPad Prism 8.0 (GraphPad Software, La Jolla, CA, USA), and SPSS (IBM, Armonk, NY, USA) were used for the other statistical analyses.

The study was approved by the Swedish Ethical Review Authority (DNR 2013/480, 2020/04750), was performed in accordance with the Declaration of Helsinki, and all study participants gave their written consent to take part in the study.

## Results

Ten subjects were included and undertook both visits. Their preoperative weight was 108.0 kg (interquartile range (IQR) 29.0), and BMI 41.3 m/kg^2^ (IQR 4.8). All subjects had undergone laparoscopic RYGB at the Uppsala University Hospital Surgical Department approximately one year before inclusion. Anthropometric measurements and preoperative values for reference, can be viewed in Table 1.

**Table 1:** Subject characteristics and fasting blood values at first study visit one year after RYGB

Subject characteristics	First study visit
N	1 M/9F
Age (years)	42 (15)
Weight (kg)	80.7 (35.8)
BMI (kg/m^2^)	30.9 (9.0)
Waist circumference (cm)	94.5 (18.5)
Hip circumference (cm)	108.5 (19.8)
Waist/Hip ratio	0.85 (0.07)
Total body fat (%)	37.1 (10.8)
BP systolic (mmHg)	122 (20)
BP diastolic (mmHg)	82 (16)
P-glucose (mmol/L)	5.2 (0.3)
HbA1c (mmol/mol)	33.3 (4.8)
P-cholesterol (mmol/L)	3.6 (1.1)
P-HDL-cholesterol (mmol/L)	1.4 (0.5)
P-LDL-cholesterol (mmol/L)	2.0 (1.5)
P-triglycerides (mmol/L)	0.8 (0.3)

*BP*  blood pressure, *P*  plasma. Data are median (IQR)

### Glucose

All subjects were normoglycemic at fasting baseline, with levels between 4.4 and 5.6 mmol/L (median 5.25 mmol/L), and glucose levels dropped below baseline after the meal for all subjects during both SAL and EXE infusions. This occurred at median 90 min in EXE and median 105 min in SAL infusion tests. Nadir glucose was below 3.0mmol/L for two subjects in SAL at 150 min, and for nine subjects in EXE 105 min or later. There were no significant differences regarding baseline, maximum, or nadir glucose or slope up to maximum glucose from baseline glucose. There was however a difference in slope down, with a faster drop in EXE group to nadir from maximum glucose. Further details are presented in Table 2.

**Table 2:** Median glucose levels (IQR) in mmol/L during standardized high carbohydrate liquid meal test

	SAL	EXE
Baseline Glucose, mmol/L	5.3 (0.5)	5.3 (0.2)
Maximum Glucose, mmol/L	10.3 (2.3)	10.4 (2.3)
Nadir Glucose, mmol/L	4.0 (0.5)	2.7 (1.2)
Slope up, mmol/L/min	0.17 (0.1)	0.11 (0.1)
Slope down, mmol/L/min	0.04 (0.0)	0.10 (0.0)*

Slope up time saline = 30 min, exenatide = 45 min. Slope down time saline = 180 min, exenatide = 120 min. *= *p* < 0.05

**Fig. 1:**
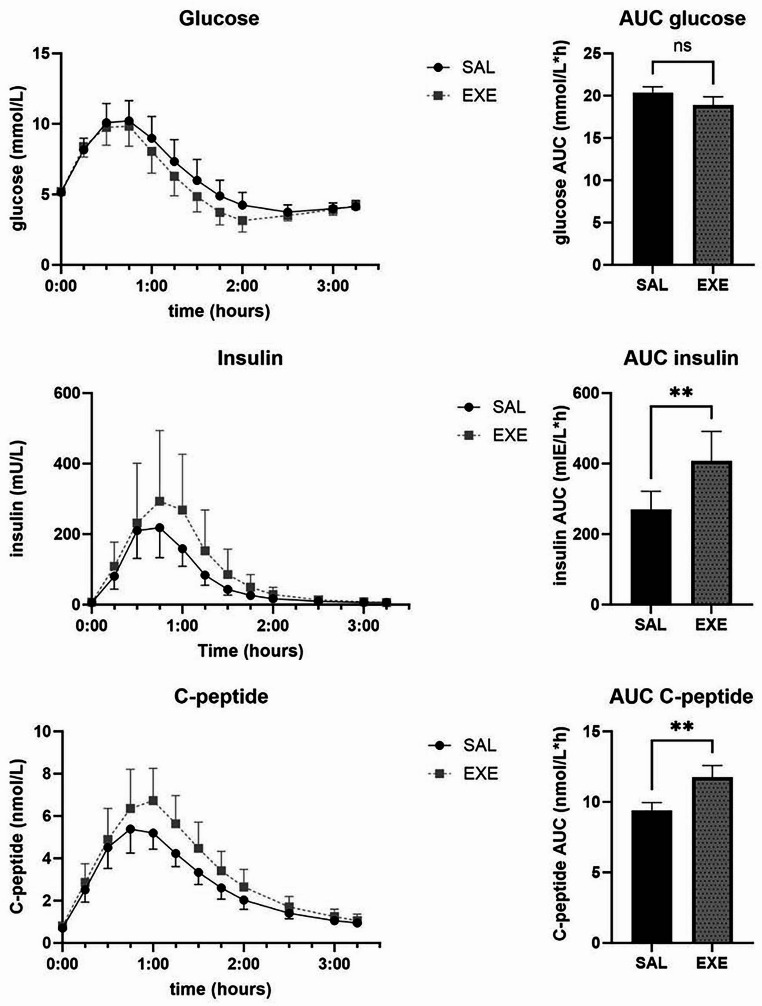
Glucose, insulin, C-peptide levels during meal test in 10 patients approx. 1 year after Roux-en-Y gastric bypass during saline (SAL) or exenatide (EXE)infusion. Values are geometric means + 95% confidence intervals. AUC = Mean total area under the curve calculated with trapezoidal rule + SEM. NS = not significant, **p < 0.01

The results of the meal test on glucose, insulin and c-peptide over time (geometric mean + 95%CI) as well as mean total AUC (± SEM) are presented in Fig. 1. Mean AUC for insulin was significantly higher for EXE than for SAL (407.5 ± 83.9 vs. 269.5 ± 51.9 mIE/L$\:\times\:$h, *p* = 0.0059). Mean AUC for C-peptide was significantly higher for EXE than for SAL (11.8 ± 0.8 vs. 9.4 ± 0.6 nmol/L$\:\times\:$h, *p* = 0.0020).

**Fig. 2:**
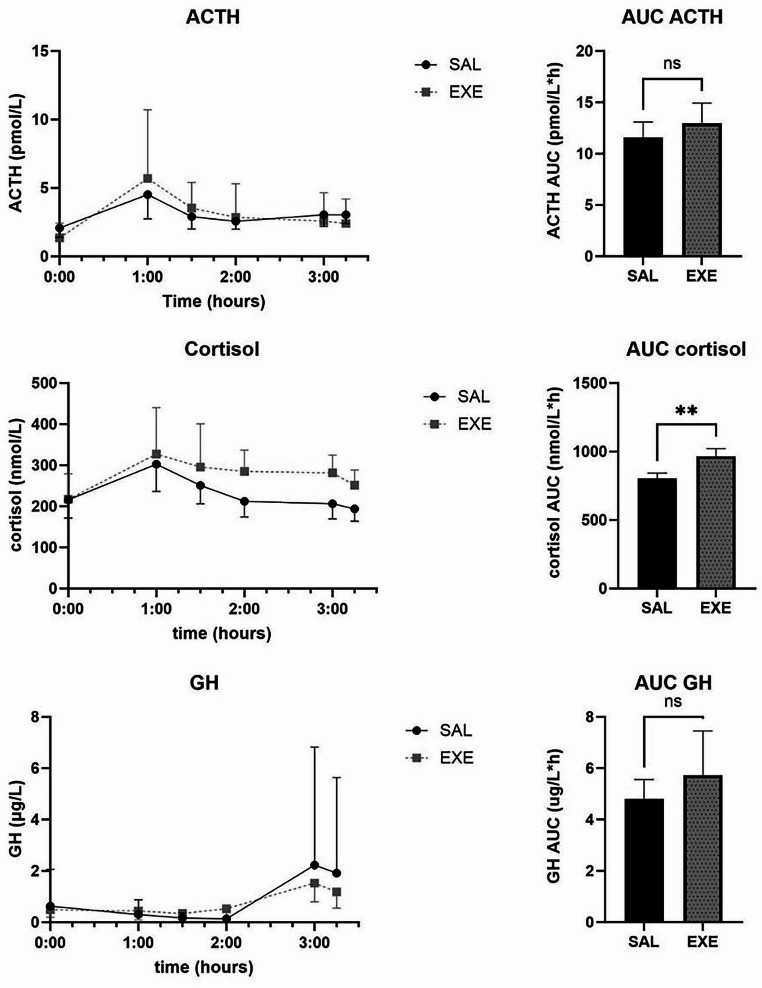
Cortisol, ACTH and GH levels during meal test in 10 patients approx. 1 year after Roux-en-Y gastric bypass during saline (SAL) or exenatide (EXE) infusion. Values are geometric means + 95% confidence intervals. AUC = Mean total area under the curve calculated with trapezoidal rule + SEM. NS = not significant, **p < 0.01

The response of cortisol, ACTH, GH and glucagon during meal test are presented in Fig. 2. There was a rise in ACTH at minute 60 for both SAL and EXE, but there was no significant difference in mean AUC. We observed a larger mean total AUC of cortisol during EXE infusion compared to SAL (966.9 ± 54.9 vs. 805.5 ± 37.7 nmol/L$\:\times\:$h, *p* = 0.0039).

**Fig. 3:**
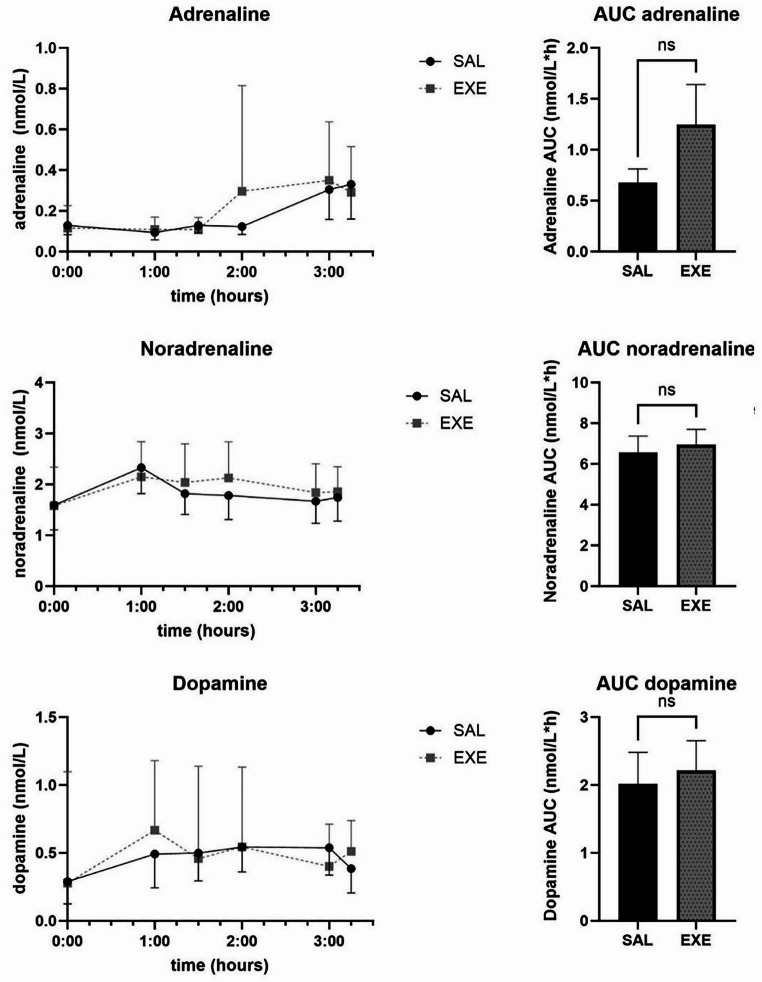
Adrenaline, noradrenalin and dopamine levels during meal test in 10 patients 1 year after Roux-en-Y gastric bypass during saline (SAL) or exenatide (EXE) infusion. Values are geometric means + 95% confidence intervals. AUC = Mean area under the curve calculated with trapezoidal rule + SEM. NS = not significant

**Fig. 4:**
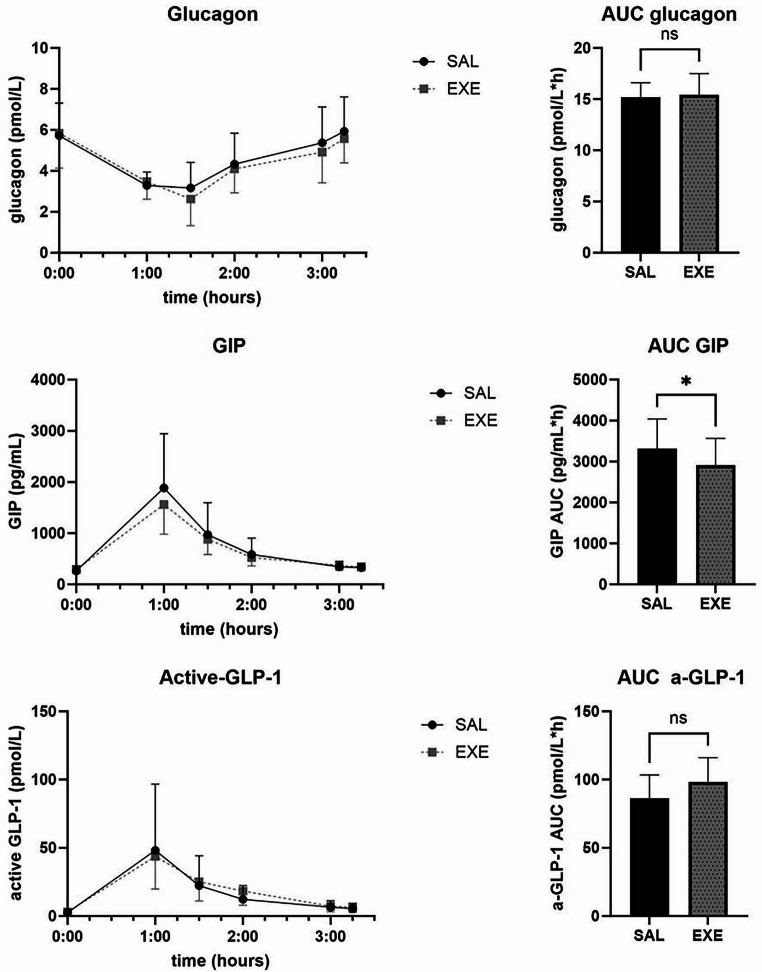
Glucagon, GIP and active GLP-1 levels during mealtest in 10 subjects 1 year after Roux-en-Y gastric bypass during saline (SAL) or exenatide (EXE) infusion. Values are geometric means + 95% confidence intervals. AUC = Mean area under the curve calculated with trapezoidal rule + SEM. NS = not significant. *p < 0.05

Levels of catecholamines adrenaline, noradrenaline and dopamine are presented in Fig. 3. Glucagon, GLP-1 and GIP curves and total AUCs are presented in Fig. 4. There were no significant differences between SAL and EXE on mean AUCs for any of these hormones.

### Paracetamol

**Fig. 5:**
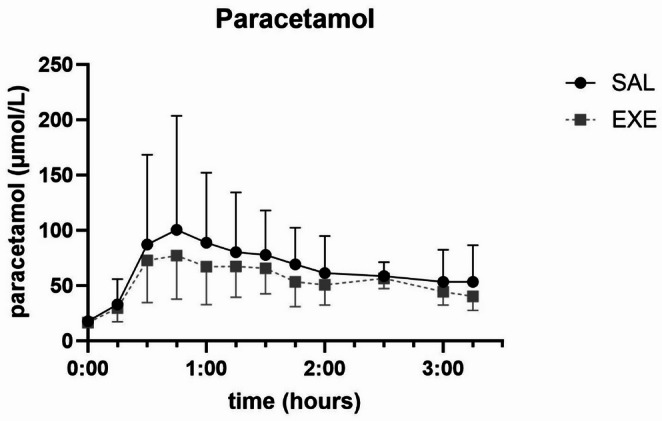
Plasma paracetamol levels after administration of 1.5 g paracetamol per os before meal test in 10 patients 1 year after Roux-en-Y gastric bypass during saline or exenatide infusion. Values are geometric means + 95% confidence intervals

Plasma paracetamol levels are displayed in Fig. 5. We found no significant differences in AUC of paracetamol between SAL and EXE.

### Heart rate variability

**Fig. 6:**
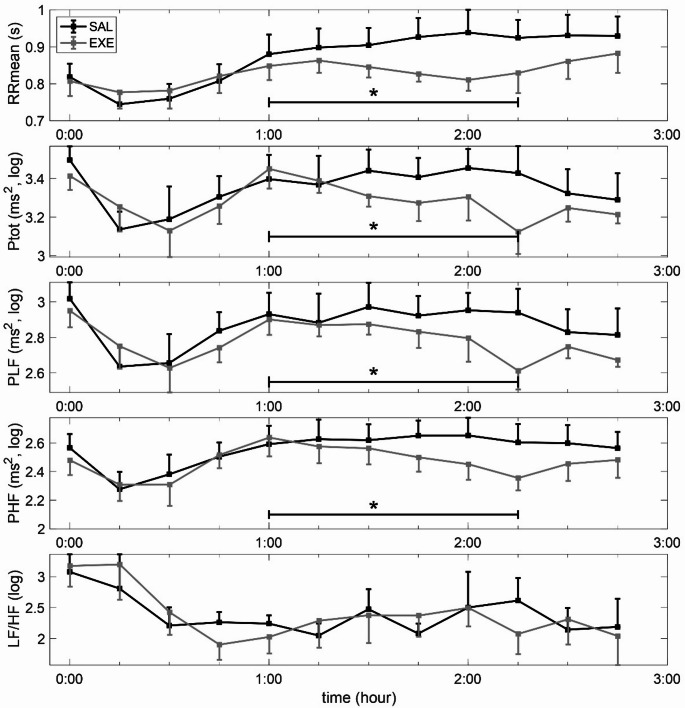
Heart rate variability recordings during meal test determined by power spectrum analysis, consecutive 15-min segments averages. Values are mean and SEM. RR = mean RR interval, P_tot_=total power, P_LF_=power of low frequency component, P_HF_= power of high frequency component. LF/HF = ratio of low frequency over high frequency component. *p < 0.05

Results from heart rate variability readings are presented in Fig. 6 which shows the mean (SEM) for different 15 min segments. No differences were noted before 60 min, after which there was a near linear change during both SAL (increase) and EXE (decline) until minute 135 when the curves tended to converge. In linear mixed effects modelling of the period 60–135 min, there was a statistically significant interaction between condition (EXE vs. SAL) and time for parameters RR (*p* = 0.01), Ptot (*p* = 0.002), PLF (*p* = 0.01) and PHF (*p* = 0.02). Since PLF and PHF where affected in the same direction the PLF/PHF-ratio remained largely unchanged for both conditions.

## Discussion

The present study of ten subjects one year after RYGB compared the effects of GLP-1 analogue exenatide to saline on the hormonal response to a liquid high carbohydrate meal test to explore potential mechanisms behind the previously reported protection against post-bariatric hypoglycemia [11]. Within the current experimental setting however, exenatide did not offer protection against post-prandial glucose declinations, not withstanding a stimulatory effect on post-prandial cortisol secretion. Although none of the participants reported symptoms of hypoglycemia, the majority exhibited postprandial glucose levels < 4.0 mmol/L under both experimental conditions, supporting the notion that most post-RYGB patients experience a state of relative postprandial hypoglycemia, possibly reflecting a lowered glucose setpoint.

Interestingly, EXE further increased insulin levels, also reflected in a faster Slope down from maximum to nadir glucose, suggesting that the GLP-1-receptor system is not fully saturated despite the already elevated endogenous GLP-1 levels after RYGB. This observation is consistent with the fact that GLP-1-receptor analogue treatment remains effective for weight loss medication following bariatric surgery, albeit not as efficiently as in non-operated individuals [16]. These findings raise the possibility that GLP-1 receptor agonists could also have therapeutic utility in the setting of recurrent diabetes after bariatric surgery, warranting further investigation.

Postprandial cortisol increases have been reported in healthy individuals [17] and recent evidence by our research group suggest that bariatric surgery leads to an upregulation of the post-prandial response of cortisol after an oral glucose load, as well as many other counter-regulatory hormones [18]. Since GLP-receptor activation in the post-RYGB state presently led to an increase of post-prandial cortisol secretion, it is possible that the accentuated endogenous GLP-1 secretion following RYGB partially explain some of these findings [19]. In further support of this concept, stimulatory effects of acute administration of GLP-1 on the HPA axis have been demonstrated in both rodents and humans. Gil-Lozano et al. reported a potent cortisol increase in fasting healthy subjects (*n* = 6) who were given GLP-1 (7–36) (1 µg/kg) intravenously. This effect of GLP-1 does not seem to be secondary to insulin-mediated lowering of glucose, as a similar stimulatory effect of GLP-1 (7–36) on cortisol secretion was seen in insulin-deficient patients with type 1 diabetes (*n* = 6) [20]. In contrast to the post-prandial setting, post-RYGB patients have been demonstrated to have a generally dampened counter-regulation, including lessened cortisol secretion, during clamped hypoglycemia [21]. Further, these findings are congruent with the accentuated hypoglycemic response of the HPA-axis that has been reported in overweight and obese individuals [22]. In fasting conditions, investigations of how the cortisol axis is affected by overweight or obesity have shown highly inconsistent results [23]. The underlying mechanisms as well as the physiological consequences of the observed dimorphic and situation specific alterations in the regulation of the HPA-axis clearly warrants further investigations.

Interestingly, the cortisol results from the current study differ from the results with exenatide given during hyperinsulinemic-hypoglycemic clamps, where no difference was observed in cortisol levels when compared to saline [12]. The lower nadir and faster rate of glucose decline in EXE group, even though not significant, might have influenced this. One other possible explanation for this discrepancy is that the effect of clamped hypoglycemia on cortisol release is large enough to mask any differential effects of EXE vs. SAL. Nevertheless, increased postprandial secretion of cortisol and other counter-regulatory hormones in the normal post-RYGB state, as shown previously [18], could contribute to the defence and/or recovery from PBH.

Regarding HRV, EXE decreased both PLF and PHF continuously during the period 60–135 min. It is commonly acknowledged that PLF reflects both sympathetic and parasympathetic tone, whereas PHF is considered to directly reflect parasympathetic activity [15]. Using a ratio of the two, PLF/PHF, can be indicative of the relative tone of sympathetic activity on the heart [24]. The findings from our previously clamp study [12] suggested an attenuation of the sympathetic in relation to the parasympathetic activity observed during GLP-1 receptor activation with EXE, both during normoglycemia and hypoglycemia, as reflected by PLF/PHF. The results from the present study show an expected effect of EXE on the RR-interval, i.e. an increased heart rate, but no change in PLF/PHF-ratio. The heart rate greatly affects other heart rate variability parameters, which could account for at least part of the observed differences between conditions in our experiment. Taken together, the present HRV findings are likely explained by a direct effect of GLP-1 activation on the sinus node, rather than through modulation of ANS activity, which is fully compatible with previous work by Lubberding et al. [25].

Strengths of this study include the randomized cross-over design, the implementation of a physiologically relevant intervention and the multimodal assessments that were undertaken.

One limitation of this study is that we chose to investigate subjects from the general post-RYGB population, rather than patients with diagnosed PBH, which decreases the generalizability towards the group with reported PBH. However, a significant proportion of patients that have undergone RYGB have asymptomatic hypoglycemias [13, 26], so we deemed it important to initially investigate responses in the typical post-RYGB state. Notably, both SAL and EXE meal tests were in fact associated with a steep peak and then decline in glucose levels, with some subjects’ values reaching the hypoglycemic range, even though none of the subjects included had previously communicated of symptoms of post-bariatric hypoglycemia. Furthermore, we did not assess hypoglycemia symptoms, which would have added important information. Further, the small sample size leads to a potential sample bias, which might influence the outcome and reduce the generalizability of our results.

In conclusion, these findings motivate further studies on subjects with reported PBH post-RYGB to elucidate the effect of GLP-1 receptor analogues during hypoglycemia. Not the least since the GLP-1 receptor antagonists are not yet commercially available, and the GLP-1 receptor agonist are probably the best currently available treatment option. In future studies it would be valuable to add tracer methodology to detect glucose kinetics and thereby more thoroughly investigate how glucose is handled in PBH and its counterregulation. Not the least since these findings demonstrate a possible effect of GLP-1 analogue treatment on postprandial cortisol secretion.

## Data Availability

No datasets were generated or analysed during the current study.
